# Lifesaving, cost-saving: Innovative simplified regimens for drug-resistant tuberculosis

**DOI:** 10.1371/journal.pgph.0001287

**Published:** 2022-11-16

**Authors:** Aastha Gupta, Sandeep Juneja, Suvanand Sahu, Mohammed Yassin, Grania Brigden, Eliud Wandwalo, Saurabh Rane, Fuad Mirzayev, Matteo Zignol

**Affiliations:** 1 TB Alliance, New York, NY, United States of America; 2 Stop TB Partnership, Geneva, Switzerland; 3 The Global Fund, Geneva, Switzerland; 4 Survivors Against TB, Mumbai, India; 5 World Health Organization, Geneva, Switzerland; McGill University, CANADA

## Introduction

Tuberculosis (TB) is one of humanity’s oldest and deadliest pandemics, accounting for an estimated 10 million cases and 1.5 million deaths globally each year [1]. Drug-sensitive TB (DS-TB) can be treated effectively and inexpensively with a six-month, four-drug treatment regimen. Drug-resistant TB (DR-TB), however, represents roughly 5% of TB cases [1], but has traditionally required outsized human and financial resources to treat, placing tremendous burden on already overtaxed patients, families, health systems, governments, and other payers.

Before recent innovations in DR-TB therapy, conventional DR-TB treatment often required 5–7 drugs and more than 14,000 pills over a duration of up to 18 months, or sometimes longer [2]. Shorter DR-TB treatment regimens of 9–11 months have also been recommended recently, but uptake by health systems has been modest. Of those who are able to access therapy, more than 40% were unable complete it successfully [1] due in part to the lengthy and complex treatment that presents significant challenges both to compliance and to healthcare systems who must administer the therapy and follow up with patients for up to an additional 18 months or more.

The recently developed BPaLM and BPaL drug regimens (BPaLM/BPaL) have demonstrated success rates of approximately 90% among people with multidrug- or rifampicin-resistant tuberculosis (MDR/RR-TB) and pre-extensively drug-resistant tuberculosis (pre-XDR-TB) in clinical research studies [3–5]–the drug-resistance profiles that represent the vast majority of the DR-TB burden. These regimens hold promise to improve treatment outcomes and experiences in these groups of historically difficult-to-cure patients, simplify care for patients at multiple levels throughout the cascade of care, and improve the currently low treatment success rates. If scaled, there is now potential to align the duration and management of drug-sensitive and drug-resistant forms of TB for the first time in the history of TB treatment. Doing so could alleviate many significant constraints and costs typically related to the management of most forms of drug resistant TB.

The all-oral, 6-month BPaLM/BPaL regimens comprise three to four drugs: the new drug pretomanid (developed by the non-profit TB Alliance), used in combination with bedaquiline and linezolid, with or without moxifloxacin. The World Health Organization, in its May 2022 Rapid Communication has informed that BPaLM may be used programmatically for all people with rifampicin-resistant TB who are ≥14 years and have not had previous exposure of >1 month to bedaquiline, pretomanid and linezolid, while moxifloxacin may be dropped in case of known resistance to fluoroquinolones (i.e., pre-XDR-TB). BPaLM/BPaL may be used in place of the previously recommended 9–11 months shorter treatment regimen (STR) and 18–24 months longer treatment regimens (LTR) [6]. The evidence from the available studies suggests that these regimens may be used in eligible patients with MDR/RR-TB and pre-XDR-TB regardless of their HIV status. Further, The Global Fund has indicated willingness to support countries to transition to this regimen.

The need to reduce the cost of treatment has historically been a substantial obstacle to scaling up treatment for most forms of drug resistant TB. The authors have calculated an estimated potential savings if the new regimen is implemented. However, they recognize that a more detailed approach would better enable the modeling of true savings from rapidly adopting and scaling up access to the BPaLM/BPaL regimens, in consideration of complex country-by-country differences. The authors also recognize that improved availability of drug susceptibility testing including rapid molecular tests and line probe assay and enhanced active drug safety monitoring and management (aDSM) will be an important factor to help scale up BPaLM/BPaL regimen and encourage early and rapid deployment of the same.

## New regimens are projected to lower the cost of treating drug-resistant TB

The main drivers of treatment costs for drug-resistant TB are medicines, health systems costs (which include diagnostics and patient follow-ups), and patient-incurred costs.

### Medicine costs

Based on prices listed on the Global Drug Facility’s Product Catalog, treatment with STR costs $545-$660 per treatment episode, and LTR costs $875-$945. BPaLM/BPaL regimens may cost $720-$725 at the present low volumes [7]. Hence while BPaLM/BPaL combination is 9–32% more expensive than STR, it is 18–23% cheaper than LTRs, with a potential to become cheaper with higher volumes.

### Healthcare costs

Healthcare costs, which include costs incurred by health systems, render the current STR and LTR regimens expensive for programmes to implement at the scale required. A publication led by the developer of bedaquiline presented the total cost of treatment (excluding patient costs) in three high MDR/RR-TB burden countries (South Africa, Russian Federation and India). When using BPaLM/BPaL regimens, the potential savings can be estimated at ~40% compared to STR (~$1,000-$2,000 savings per patient) and ~75% compared to LTR ($4,000-$6,000 savings per patient) [8]. Several other publications modelling costs of treatment for people with MDR-TB and pre-XDR, or who are MDR-TB treatment intolerant or nonresponsive, estimate the savings on using BPaL would range between 80% to 90% (up to $12,000 per patient) [9, 10]. These significant savings would primarily result from the shortened duration of treatment and the resulting lower healthcare costs, including fewer monthly follow-ups and a reduced need for lab-based treatment monitoring.

Extrapolating an average of these numbers to understand the potential scale of benefit shows that savings could reach $740 million annually if all patients were to transition to BPaLM/BPaL immediately (assuming similar scales of enrollment of MDR/RR-TB patients [121,228] and XDR-TB patients [22,029] as reported in the Global TB Report 2021) [11]. This cost saving by health systems for care to patients undergoing treatment would be in addition to the savings in patient-incurred costs. Even without any increase in available resources, these savings could fund MDR/RR-TB treatment for additional ~400,000 patients or drug-susceptible TB treatment for ~3.1M patients [12].

### Patient-incurred costs

Additional cost savings associated with implementing the BPaLM/BPaL regimens stem from a reduction of costs incurred by patients, including travel, nutrition and most importantly, the loss of productivity due to the inability to work while completing treatment. In 19 surveyed countries, WHO reported that 87% of drug-resistant TB patients and their households experienced catastrophic total costs (defined as >20% of annual household income) [13]. The new regimens have the potential to significantly shorten the period of income loss and reduce the costs faced by patients.

Further, patients not only incur monetary costs, but suffer various other opportunity costs. Drug resistant TB can cause an array of physical and mental issues, that result patients foregoing time, professional development, and quality of life. Many young patients enrolled in school are forced to interrupt their studies for an extended period due to which their career growth is often stunted. When a patient is the primary financial earner for a family, entire families are affected due to the nature of the disease and treatment. DR-TB Patients have consistently iterated two central asks to improve their DR-TB treatment experience—faster time to diagnosis and shorter treatment regimens. The value of a shorter treatment is lies not only in reducing suffering but also giving patients back their time, an invaluable resource.

## Conclusion

It is widely believed that new treatments tend to be more expensive than existing therapies that have been used for many years and have gained scale in the market. However, the cost of implementing BPaLM/BPaL regimens, even without accounting for patient-incurred costs, is potentially 40–90% less expensive when compared with current regimens, despite containing two innovative new drugs (bedaquiline and pretomanid). In addition to the cost savings, the BPaLM/BPaL regimens significantly reduce the pill burden and economic hardship for patients, simplifying administration and improving the patient experience. The STR requires 13–14 pills per day for 9–11 months and the LTR 4–5 pills for 18–24 months [7]. Comparatively, the BPaLM/BPaL regimens require, on average, 3–4 pills per day for just 6 months.

The development of these new regimens has the potential to transform drug-resistant TB treatment, benefiting patients and health systems globally. Countries should prioritize the implementation and scale up of BPaLM/BPaL regimens as there is 1) no financial barrier to procurement; 2) programmatic and patient costs promise cost savings; 3) clinical and operational research shows improved treatment outcomes compared to current standards of care; and 4) the transition to these regimens is supported by standard setting bodies and funding organizations like WHO and the Global Fund.
